# Combined molecular characterization and dopa-responsive treatment in two patients with NR4A2-associated intellectual developmental disorder

**DOI:** 10.3389/fgene.2025.1590292

**Published:** 2025-09-11

**Authors:** Na Liang, Ting Li, Yang Deng

**Affiliations:** Department of Medical Genetics and Prenatal Screening, Taiyuan Maternal and Child Health Hospital, Taiyuan, China

**Keywords:** NR4A2 gene, intellectual developmental disorder, dopa-responsive dystonia-parkinsonism, whole-exome sequencing, RNA sequencing, L-DOPA treatment

## Abstract

**Introduction:**

Pathogenic variants in *NR4A2* are associated with neurodevelopmental disorders including intellectual developmental disorder with language impairment and early-onset dopa-responsive dystonia-parkinsonism (IDLDP). Here we report two pediatric *NR4A2*-related cases presenting with global developmental delay, speech impairment, and intellectual disability.

**Methods:**

Comprehensive genetic investigations including whole-exome sequencing revealed a *de novo* missense variant (c.994G>C, p.Val332Leu) in *NR4A2* and a 2q23.3-q24.2 deletion encompassing *NR4A2*. Functional validation via RNA sequencing revealed that the missense variant induces pathogenic exon 4 skipping through aberrant splicing. Both patients exhibited marked clinical improvements in linguistic competence and motor function following levodopa therapy, initiated after confirmation of dopaminergic responsiveness. A systematic review of 19 reported *NR4A2*-related cases revealed substantial phenotypic heterogeneity, with three of them demonstrating favorable responses to dopaminergic treatment.

**Results:**

Our findings underscore the diagnostic value of integrating molecular profiling with functional RNA analysis to resolve complex neurogenetic disorders. Levodopa therapy shows therapeutic potential for *NR4A2*-deficient patients with dopa-responsive features, especially in linguistic improvement. This study expands the understanding of *NR4A2*-associated pathogenesis and provides insights for the precision management of related neurodevelopmental conditions.

## 1 Introduction

*NR4A2* gene (MIM *601828), also known as *NURR1*, encodes nuclear receptor subfamily 4 group A member 2, a member of the steroid–thyroid hormone–retinoid receptor superfamily, which is a conserved transcription factor implicated in the development and maintenance of dopamine-synthesizing cells. By regulating gene expression in the human brain, *NR4A2* specifically influences the maturation and development of neurons, particularly dopaminergic neurons (Lévy et al., 2018). Structural and sequence variants in *NR4A2* leading to haploinsufficiency have been identified in patients with intellectual developmental disorder characterized by language impairment and early-onset dopa-responsive dystonia-parkinsonism (IDLDP), with the first reports published in 2017 (Reuter et al., 2017). Phenotypic heterogeneity exists and the severity is highly variable, but core symptom of patients is developmental delay affecting motor, cognitive, and speech, with half of patients develop various types of seizures (Gabaldon-Albero et al., 2024). Since *NR4A2* plays a role in maintenance of adult midbrain dopaminergic neuron, it is also associated with neurodegenerative diseases, such as Parkinson’s or Alzheimer’s disease, schizophrenia, and manic depression (Le et al., 2003; Buervenich et al., 2000).

Following comprehensive clinical evaluations of two pediatric cases (an 8-year-old and a 6-year-old) presenting with global developmental delay with predominant language impairment and intellectual disability, whole-exome sequencing (WES) revealed a missense variant and a microdeletion involving *NR4A2* gene. The patient with the missense variant underwent further functional validation through RNA sequencing of peripheral blood specimens, which demonstrated the missense variant’s disruptive effect on splicing fidelity, culminating in a definitive molecular diagnosis. Both patients demonstrated marked improvement in linguistic competence and motor function following initiation of levodopa therapy, which was instituted after confirming dopaminergic responsiveness through clinical trials.

In-depth phenotypic characterization, detailed treatment protocols, and a comprehensive review of published *NR4A2* cases offer a thorough understanding of patients afflicted with intellectual developmental disorder with language impairment and early-onset DOPA-responsive dystonia-parkinsonism. Furthermore, exploration of the molecular mechanisms underlying *NR4A2*-associated intellectual developmental disorder with language impairment and early-onset DOPA-responsive dystonia-parkinsonism is imperative for the development of efficacious therapies and enhanced clinical outcomes.

## 2 Methods

### 2.1 Clinical evaluation of the patient

The evaluation of two pediatric patients with intellectual disability and language delay involved a multidisciplinary approach, including medical history review, clinical examination, and neurodevelopmental assessments. A comprehensive medical history was obtained for both patients, focusing on developmental milestones (motor, cognitive, and language domains), family history of neurodevelopmental disorders, and prenatal/perinatal complications. A detailed physical examination was conducted to assess anthropometric measurements and identify dysmorphic features. Intellectual functioning was measured using the Wechsler Intelligence Scale for Children (WISC-V), while language abilities were evaluated with International Classification of Functioning, Disability and Health (ICF) Speech Function Assessment Criteria. Dopamine and homovanillic acid levels in cerebrospinal fluid (CSF) was measured for prediction of dopa-responsive.

### 2.2 Whole exome sequencing and analysis

Trio-based whole-exome sequencing (WES) was conducted to identify any underlying genetic causes. In brief, genomic DNA was extracted from the patient’s peripheral blood and fragmented into 150–200 bp fragments. Sequencing libraries were constructed utilizing the SureSelect XT Human All Exon V6 kit from Agilent Technologies (Santa Clara, CA, United States), and sequencing was carried out on the Illumina NovaSeq 6000 System (Illumina, San Diego, CA, United States). Data quality control and adaptor sequence removal were performed using Fastqc (Babraham Research Institute, Cambridge, United Kingdom) and Fastp (Visible Genetics, Inc., Toronto, Canada) tools, respectively. Alignment with the reference genome was conducted utilizing SpeedSeq (Ira Hall Lab, St. Louis, MO, United States). The Genome Analysis Toolkit (Broad Institute, Cambridge, MA, United States) was employed to identify variations in the BAM file meeting quality control criteria, generating a VCF format file. Annotation of variations in VCF files was carried out using the Ingenuity Variant Analysis (Ingenuity Systems, Redwood City, CA, United States) and Translational Genomics Expert platforms. All potential variants were confirmed through Sanger sequencing and validated using parental test results. Copy number variants were detected using the CNVkit open-source software (Larsen et al., 2016). Pathogenicity classification performed according to the American College of Medical Genetics and Genomics (ACMG) guidelines. Sanger sequencing was used to confirm any identified variants. The clinical findings were correlated with genetic data to form a comprehensive diagnostic profile.

### 2.3 RNA-sequencing and data analysis

The patient’s peripheral blood was drawn and total RNA was extracted by using the RNeasy Kits (Qiagen). Poly(A) mRNA was selected and the complementary DNA (cDNA) libraries were prepared with the Illumina TruSeq Stranded mRNA Library Prep kit (Agilent, Santa Clara, CA, United States) following the manufacturer’s instructions. The cDNA libraries were sequenced on an Illumina NovaSeq 6000 System (Illumina, Inc., San Diego, CA, United States) using 150 bp paired-end reads.

The generated FASTQ files underwent quality control and adapter sequence removal using Fastqc v.0.11.9 (Babraham Research Institute, Cambridge, United Kingdom) and Fastp v.0.20.1 (Visible Genetics, Inc., Toronto, Canada), respectively. To detect gene fusions and enhance the sensitivity of novel splice junction detection, clean data were aligned to the reference human genome (hg19) using STAR v.2.7.8a (Cold Spring Harbor, New York, United States) with the parameters twopassMode and chimeric output function (chimSegmentMin = 12, twopassMode = ‘Basic’). Gene-level quantifications were performed using RSEM (v1.2.28). Differentially expressed genes (DEGs) were identified compared to controls with an adjusted p value <0.05 cutoff (q-value) using DESeq2 (v.1.26.0). LeafCutter was employed to assess the statistical significance of differences in the quantity of each splicing event (minclureads = 30; maxintronlen = 500,000; mincluratio = 1e-5). Specifically, each patient was compared against all other controls for differential splicing analysis (min_samples_per_group = 1; min_samples_per_intron = 1). The resulting P values were subjected to correction for multiple testing using a family-wise error rate approach. Additionally, aberrant splicing events were visualized using Sashimi plots generated with MISO (v.0.5.4) and Integrative Genomics Viewer (IGV). STAR-Fusion utilized chimeric and discordant read alignments identified by the STAR aligner to predict fusion events.

## 3 Result

### 3.1 Clinical manifestation

Patient 1, an 8-year-old male, was born at full-term via spontaneous vaginal delivery, with no complications noted during the labor examination. He has a 22-year-old sister who is developing normally. At 19 months of age, the patient was first referred for medical evaluation due to concurrent delays in language and motor development. When assessed using the Wechsler Intelligence Scale for Children-Fourth Edition (WISC-IV) at the age of 8, his Full Scale IQ (FSIQ) score was 65, which is indicative of a significant intellectual development disorder. The assessment based on the International Classification of Functioning (ICF) revealed mild impairments in speech and language development.

Patient 2, a 7-year-old female, was born at full-term via cesarean section. Prenatal ultrasound revealed oligohydramnios (amniotic fluid index [AFI] <5 cm). She has a 12-year-old brother with normal development. Her FSIQ score on the WISC-IV was 57. Developmental assessments further indicated overall delays in language, gross motor skills, fine motor skills, and social skills.

In order to evaluate dopaminergic dysfunction, the levels of dopamine and homovanillic acid in cerebrospinal fluid were measured. For Patient 1, the dopamine level in the CSF was 10.5 ng/mL (normal range: 20–30 ng/mL), and the HVA level was 180 ng/mL (normal range: 300–600 ng/mL), indicating a significant reduction in dopaminergic activity. For Patient 2, the dopamine level was 15 ng/mL (normal range: 20–30 ng/mL), and the HVA level was 220 ng/mL (normal range: 300–600 ng/mL), also suggesting dopaminergic dysfunction. Based on these findings, L-DOPA therapy was initiated at 8.5 years for Patient 1 and at 7.75 years for Patient 2, respectively.

The therapeutic regimen strictly adhered to the guidelines for children with dopa-responsive dystonia. The initial dose was set at 0.5 mg/kg per day, divided evenly into four doses, and was gradually increased to 2 mg/kg per day.

After 6 months of continuous treatment, remarkable improvements were observed in the language and motor development of both patients. Moreover, as evaluated by the WISC-IV, the Full Scale IQ of Patient 1 and Patient 2 increased by 10 and 12 points, respectively. Additionally, parental feedback indicated substantial enhancements in the patients’ verbal and physical abilities (Supplementary Video S1).

### 3.2 Review of reported NR4A2 cases

In this study, we performed a systematic review of the pathogenic variants in the *NR4A2* gene reported in pediatric patients and collected their detailed clinical information. As of December 2023, a comprehensive literature search of PubMed, Embase, and Web of Science databases identified 10 studies documenting 19 pediatric cases with *NR4A2* gene variants, encompassing 21 unique pathogenic variants (Table 1). Among the reported pathogenic variants within the *NR4A2* gene, nine were missense variants, six were frameshift variants, two were nonsense variants, and two were splicing variants (see Table 1).

**TABLE 1:** Clinical and genetic features.

	Our cases		Case identifiera (original publication)	
	Our patient 1	Our patient 2	Review of reported NR4A2 cases	
			Below 14 (Winter et al., 2021; Ramos et al., 2019; Singh et al., 2020; Song et al., 2022)	Over 14 (Singh et al., 2020; Wirth et al., 2020; Jesús et al., 2021; Feliciano et al., 2019)
Age (years)/Sex	8/M	6/F	11	8
Motor milestones	Delayed	Delayed	Delayed (9/11)	Delayed (6/8)
Hypotonia	NA	Yes	Yes (8/11)	Yes (1/8)
ID severity	Mild	Severe	Mild (9/11)	Mild (5/8)
Language	Delayed	Delayed	Delayed (8/11)	Delayed (5/8)
Psychiatric and behavioral	NA	Yes	Yes (6/11)	Yes (5/8)
Epilepsy	NA	NA	Yes (4/11)	Yes (4/8)
Movement disorder	NA	NA	Yes (2/11)	Yes (4/8)
Dopa responsive	Yes	NA	Yes (1/11)	Yes (2/8)
MRI	Normal	Abnormal	Normal (7/11)	Normal (3/8)
Type	Missense	Deletion	Missense (5/11))	Missense (4/8)
*de novo*	Yes	Yes	Yes (10/11)	Yes (7/8)

ASD, autism spectrum disorder, IA impaired awareness; CVI, cerebral visual impairment; RWLD, reading and writing learning disorder; cMR, cerebral magnetic resonance; cCT, cerebral computed tomography; NA, not available.

In this study, we conducted an in-depth analysis of three patient cases characterized by suboptimal overall development. Following the administration of dopamine therapy, these patients exhibited notable improvements across multiple metrics (see Table 2).

**TABLE 2:** Patients with NR4A2 (likely) pathogenic variants and movement disorder symptoms reported to date.

Case identifiera (original publication)	Gender/age(y)	NR4A2 variant type (inheritance)	Neurodevelopmental comorbidity	Response of movement disorder to levodopa therapy
Present patient 1 (this study)	Male/8	Missense (*de novo*)	Developmental delay, language impairment	Symptoms dramatically improved
Present patient 2 (this study)	Female	deletion (*de novo*)	Developmental delay, language impairment, intellectual disability	Symptoms dramatically improved
Patient 3 (Winter et al., 2021)	Male/2.5	Missense (*de novo*)	Developmental delay, hypotonia, language, impairment, abnormal EEG	Significant reduction of limb dystonia
Patient 4 (Wirth et al., 2020)	Male/29	Loss-of function (*de novo*)	Developmental delay, language impairment, intellectual disability, epilepsy	Total recovery of limb dystonia and parkinsonism and a dramatic decrease of the oro-mandibular dystonic episodes
Patient 5 (Wirth et al., 2020)	Female/57	Loss-of function (unkown)	Intellectual disability	Symptoms dramatically improved

### 3.3 Identification of genomic variants in *NR4A2* gene and aberrant splicing event revealed by RNA-sequencing

**FIGURE 1:**
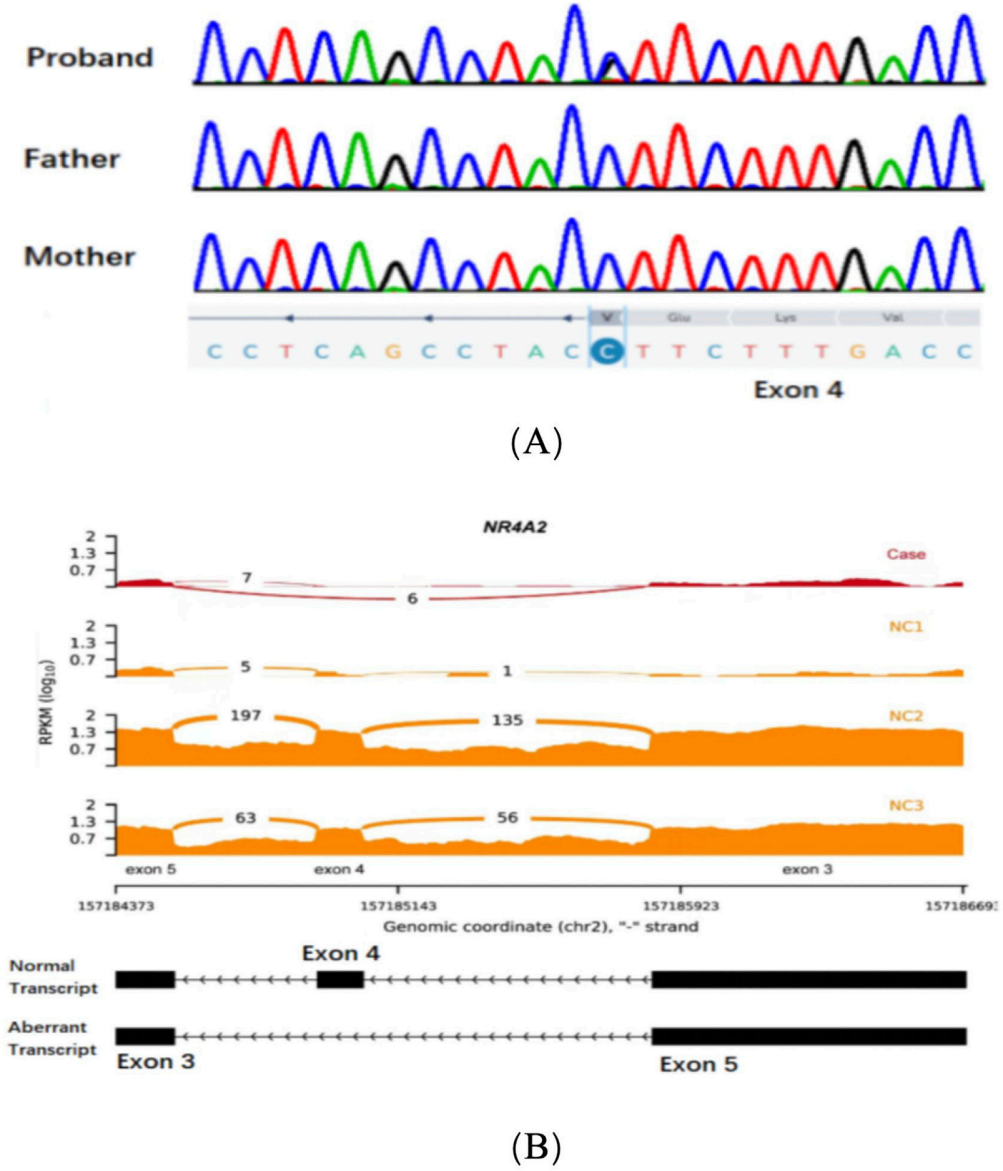
**(A)** Sanger sequencing chromatograms of exon 4 in the NR4A2 gene for the proband, father, and mother. **(B)** RNA sequencing data showing NR4A2 exon coverage. The reads per kilobase per million mapped reads (RPKM) values are plotted for three normal controls (NC1, NC2, and NC3) and the case (Case). The case shows an abnormal splicing pattern with a significant additional exon 4 skipping transcript compared to the normal controls.

Patient 1: Exome sequencing identified a missense variant, c.994G>C (p.Val332Leu) based on the reference sequence NM_006186.4 for the NR4A2 gene. Subsequently, Sanger sequencing was performed on the proband and his parents to validate the variant. The results of Sanger sequencing confirmed the *de novo* nature of this variant (Figure 1A). It was not observed in population databases (gnomAD, in-house exomes). Significantly, the SpliceAI software was employed to predict the impact of the variant carried by the patient on mRNA splicing. The predictions indicated a high level of deleteriousness, with a score of 0.9 for the c.994G>C variant. Furthermore, an aberrant splicing event associated with the missense variant within the NR4A2 gene was detected by RNA sequencing analysis. This event led to the production of an aberrant transcript characterized by the skipping of exon 4 of the NR4A2 gene (Figure 1B). In line with the American College of Medical Genetics and Genomics (ACMG) guidelines for the interpretation of genetic variants, this variant was categorized as ‘likely pathogenic’ (PS2+PM2_supporting + PP3).

**FIGURE 2:**
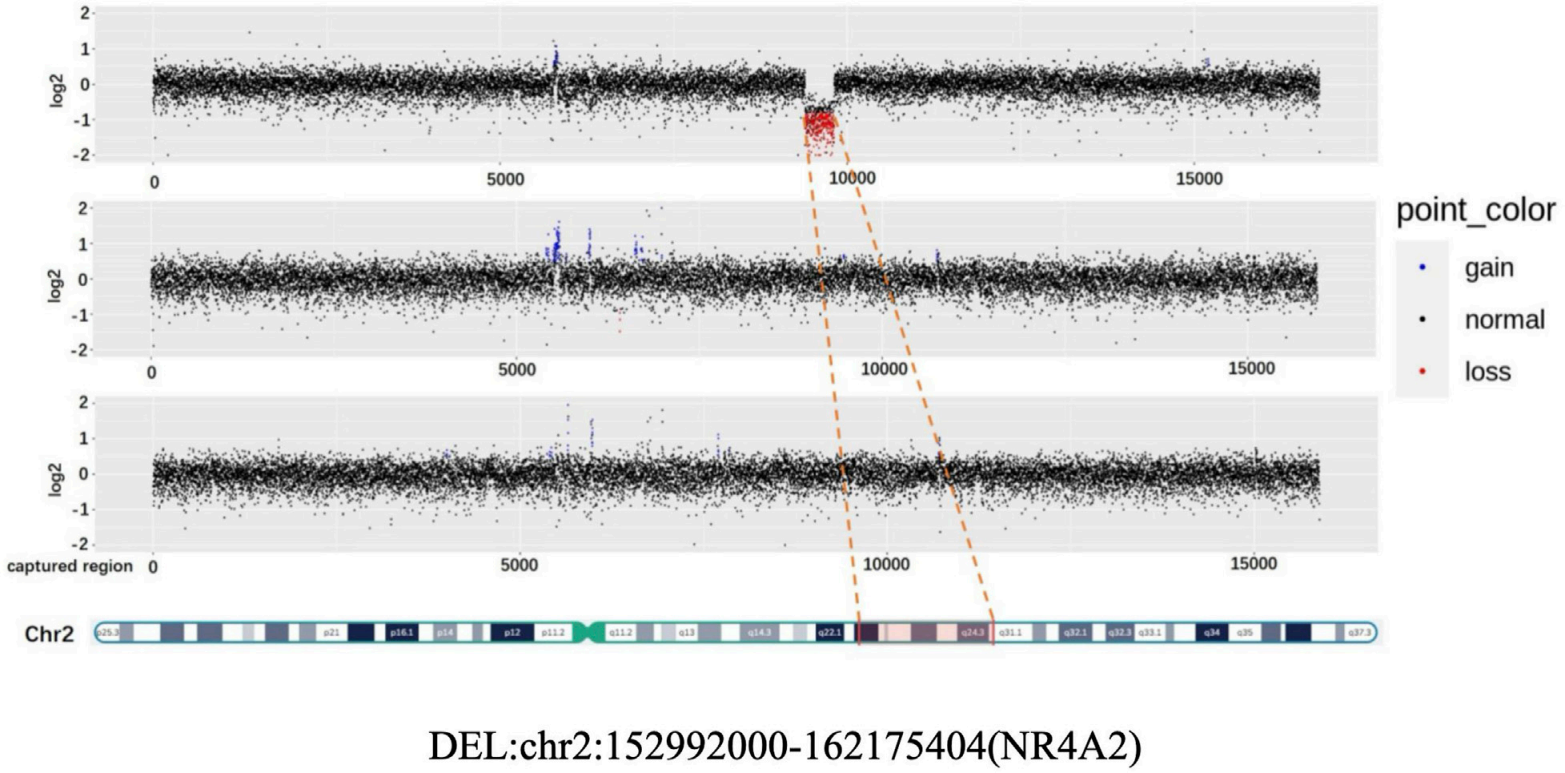
CNV (Copy Number variation) map depicting the deletion in chromosome 2 for the proband, father, and mother.

Patient 2: Copy number analysis based on WES data revealed the presence of a 9.2 Mb deletion, spanning from q23.3 to the q24.2 region on the chromosome 2. According to the ACMG guidelines, this deletion was classified pathogenic. It is worth noting that this deleted region encompasses the haploinsufficient gene *NR4A2* (Figure 2).

## 4 Discussion

In the present study, exome sequencing and genome-wide copy number variation analysis were employed to identify a missense variant and a microdeletion involving the *NR4A2* gene in two patients who exhibited global developmental delay and speech deficits. Subsequent RNA sequencing analysis was carried out to decipher the pathogenesis underlying the missense variant. According to metrics in Decipher databases (pHaplo = 0.98, pLI = 1) and clincal cases encompassing only *NR4A2* gene published by Lévy J, Grotto Set al (Lévy et al., 2018), *NR4A2* haploinsufficiency is associated with related neurological disorders. Thus transcriptional alteration derived from the novel missense variants in our patient supported its haploinsufficiency consequence, while the 9.2 Mb deletion encompassing 2q23.3 to q24.2 will inevitably lead to haploinsufficiency of *NR4A2*. The results of this analysis demonstrated abnormal splicing of the transcript, thereby shedding light on the molecular mechanism. After a comprehensive and detailed clinical evaluation of both patients, levodopa therapy was administered with the aim of addressing potential alterations in dopamine response. Remarkably, this treatment regimen resulted in substantial improvements in both language development and neurophysiological function.

*NR4A2* plays a pivotal role in the development of nigrostriatal dopamine neurons and is therefore implicated in the pathogenesis of neurodegenerative diseases linked to the midbrain dopamine system (Larsen et al., 2016). This gene, which is actively expressed during embryogenesis, plays a critical role not only in the early differentiation of midbrain dopaminergic neurons but also in the long-term maintenance of these neurons throughout adulthood. Genetic variants within this gene have been reported in patients suffering from intellectual developmental disorder characterized by language impairment, as well as early - onset DOPA - responsive dystonia - parkinsonism.

Dysfunction of the *NR4A2* gene has been linked to a wide range of disorders associated with dopaminergic impairment, encompassing Parkinson’s disease (PD), schizophrenia, and manic depression (Bannon et al., 2002). In PD, the progressive degeneration of dopaminergic neurons in the substantia nigra leads to a significant reduction in dopamine synthesis and release (Surmeier et al., 2017). This dopaminergic deficit is a key pathological feature of the disease and contributes to the characteristic motor and non-motor symptoms observed in PD patients. The dopaminergic system’s dysregulation affects the basal ganglia circuitry, disrupting the balance between direct and indirect pathways (Gerfen and Surmeier, 2011). L-DOPA, a well-recognized dopamine precursor, is capable of efficiently alleviating symptoms by restoring dopamine levels. Its efficacy has been demonstrated across a variety of diseases related with altered dopa-responsiveness (Chase, 1998).

Therefore, we have summarized here the clinical characteristics based on a total of 19 cases (Table 1). At the last clinical assessment, patient ages ranged from 2 to 32 years. No abnormalities in prenatal or perinatal history were detected in any case; growth parameters were normal. Developmental delay and/or intellectual disability was the characteristic shared by all the patients, ranging from mild to severe. Language disorder was the second most frequent, being reported in 14 patients. In one case, expressive language was absent at 6 years (Shimojima et al., 2017), while in eight patients, a moderate to severe disorder of the expressive and receptive language was noted; information on this issue was unavailable in five cases. Learning disabilities were reported in six cases, most frequently related to the acquisition of reading and writing. Neuropsychiatric issues were described in 10 patients and included aggressive behavior, anxiety, ADHD, and autism. In terms of motor development, delays were observed in nine cases, along with reports of hypotonia. Nevertheless, there were no reports of any patients being unable to achieve independent walking. In one particular case, the patient was only able to walk independently after receiving treatment with L-dopa (Winter et al., 2021).

Movement disorders were reported in seven patients. Among them, symptoms manifested during childhood in only one case, with the most common onset period occurring between late adolescence and young adulthood. The reported phenotypes included adult-onset dystonia-parkinsonism (3/19), isolated dystonia (2/19), dystonia accompanied by chorea (1/19), ataxia associated with motor tics (1 out of 19), and isolated ataxia (1/19). It is noteworthy to mention the evolutionary nature of the semiology depicted in certain of these cases. The symptoms may initiate as tics, progress to focal dystonia, and subsequently develop into dystonia-parkinsonism (Albanese et al., 2013). In one particular case, focal dystonia in the form of oculogyric crises emerged during the course of treatment with olanzapine, a dopaminergic antagonist (Haggstrom et al., 2017; Friedman, 2016). This occurrence could potentially be attributed to an augmented susceptibility to extrapyramidal symptoms associated with the utilization of such drugs. Epilepsy is also a frequent sign, reported in 42% (8/19) patients. Almost half of the patients present epilepsy, which may initiate at any age and in a diverse semiology, accompanied by the aforementioned complex and in many occasions severe neurodevelopmental disorder.

After detecting the missense variant in the NR4A2 gene through WES, according to the ACMG variant classification guidelines, were categorized as variants of Variant of Uncertain Significance (VUS). However, considering the highly concordant disease phenotype caused by this gene with the patient’s current phenotype, the variant was still considered as potential genetic etiologies. Combining the predictive results of from SpliceAI software does not rule out the possibility of this rare variants affecting mRNA splicing. The peripheral blood sample was collected as an actionable sample for RNA sequencing, given that the expression of the *NR4A2* gene was regarded as abundant. Subsequently, via RNA sequencing analysis, it was successfully determined that this rare variant directly influenced splicing, ultimately leading to an outcome analogous to that of loss-of-function variants. The variant’s pathogenicity classification was reclassified from VUS to LP following RNA-seq validation of aberrant splicing patterns, aligning with ACMG guidelines for functional evidence integration. Complementary to DNA sequencing, RNA-seq has recently been utilized to detect abnormalities in the transcriptome, such as aberrant expression, splicing, and monoallelic gene expression (MAE), thereby increasing the molecular diagnostic yield by approximately 7.5%–36% (Li et al., 2024). RNA-seq can effectively detect abnormal splicing and related variations, which can help improve the diagnosis rate of most previously undiagnosed neuromuscular diseases (Cummings et al., 2017).

Congenital dopa-responsive disorders are a group of heterogeneous conditions. Their hallmark is the alleviation of clinical symptoms upon the administration of levodopa and/or dopaminergic agents. Among the reported patients with NR4A2 variants, levodopa was prescribed to three of them for symptom management. Patient 1 presented at the age of 29 with early-onset dystonia-parkinsonism, intellectual disability (ID), and epilepsy. When his intelligence quotient was assessed at 37 years old, it was found to be 56. Levodopa treatment led to a complete recovery from limb dystonia and parkinsonism, along with a remarkable reduction in oro-mandibular dystonic episodes. Throughout the course of the disease, the daily levodopa doses required to control his symptoms gradually increased (Friedman, 2016). Patient 2 was referred at the age of 57 for a genetic diagnosis due to a phenotype characterized by a combination of dystonia, parkinsonism, and mild intellectual disability (ID). After commencing a dopaminergic treatment regimen that combined levodopa/carbidopa and bromocriptine, these symptoms showed a dramatic improvement. The third patient was a boy of German descent who presented with global developmental delay and limb dystonia that onset in infancy. When administered levodopa at a dosage of 75 mg per day, the child experienced a substantial reduction in involuntary movements. During the period when he continued to receive levodopa treatment, a trend towards accelerated motor development was noted (Friedman, 2016).

In our case, after comprehensively evaluating the severity of the patient’s clinical manifestations and the parents’ treatment preferences, we measured the levels of dopamine and homovanillic acid in the patient’s cerebrospinal fluid to assess dopamine responsiveness. Since the initiation of levodopa therapy, we have regularly monitored the patient’s intellectual function and language ability. After 6 months of treatment, the assessment revealed a five - point increase in the patient’s overall intelligence quotient. Additionally, parental reports indicated remarkable improvements in the child’s language skills and physical capabilities. Therefore, for children with similar genetic variants, when there is significant dopamine responsiveness impairment, levodopa treatment may be a viable option. However, the dosage, regimen, expected response and need for additional treatment depend on the underlying aetiology, disease severity, and adverse effects (Connolly and Lang, 2014; Hechtner et al., 2014; Gc et al., 1969; Warren Ola et al., 2013). Treatment must be tailored to the individual in collaboration with a doctor with experience in the field. The recommended starting dose of levodopa is 0.5–1 mg/kg/day. If no response is seen but the diagnosis is still suspected, the dose should be gradually increased (Connolly and Lang, 2014; Hechtner et al., 2014; Gc et al., 1969; Warren Ola et al., 2013; Willemsen et al., 2010; Echenne et al., 2006).

In conclusion, through the combined application of whole - exome sequencing and RNA sequencing, we precisely diagnosed two children carrying NR4A2 gene variants. Moreover, we pinpointed abnormal splicing transcripts as the underlying pathogenic mechanism. Following a comprehensive clinical assessment of the patients, a trial treatment with levodopa led to substantial enhancements in their overall intellectual development and language proficiency. Our experience in diagnosing and treating these cases serves as a significant addition to the existing knowledge of dopa-responsive diseases. It also offers valuable insights for the diagnosis and treatment of related neurological disorders.

Our study has several notable limitations. Enrolling only two patients significantly compromised the statistical power and generalizability of the findings for this disease cohort. Although preliminary observations of treatment response were made, large-scale multicenter cohort studies are urgently needed to validate therapeutic efficacy and elucidate the underlying molecular mechanisms. Current assessments of neurologic function recovery predominantly based on parental reports and subjective scales should be supplemented with long-term objective metrics (e.g., quantitative motor testing or neuroimaging biomarkers). A standardized long-term follow-up protocol integrating serial clinical evaluations is planned to monitor treatment durability and disease progression, with a specific focus on incorporating longitudinal data to refine prognostic models.

## Data Availability

All data analyzed during the current study are available from the corresponding author (YD) on reasonable request.
