# Supplementary figures and images for: Combined molecular characterization and dopa-responsive treatment in two patients with NR4A2-associated intellectual developmental disorder
Source: Front Genet. 2025 Sep 11;16:1590292. doi: 10.3389/fgene.2025.1590292 (PMC12460123; doi:10.3389/fgene.2025.1590292)

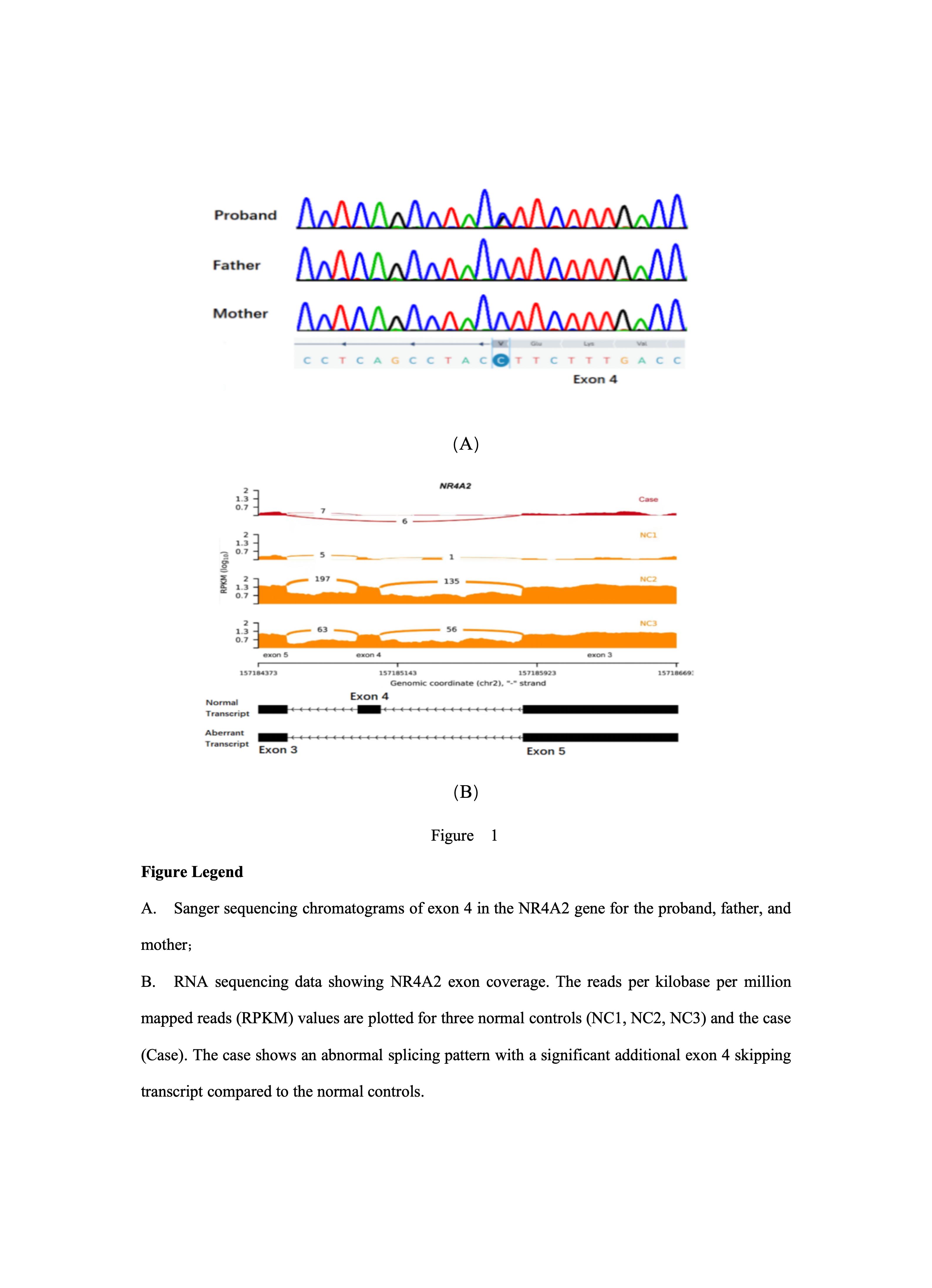

Supplement: Supplementary file 1 [file DataSheet1.zip › Supplementary files/Figure 1.jpg]

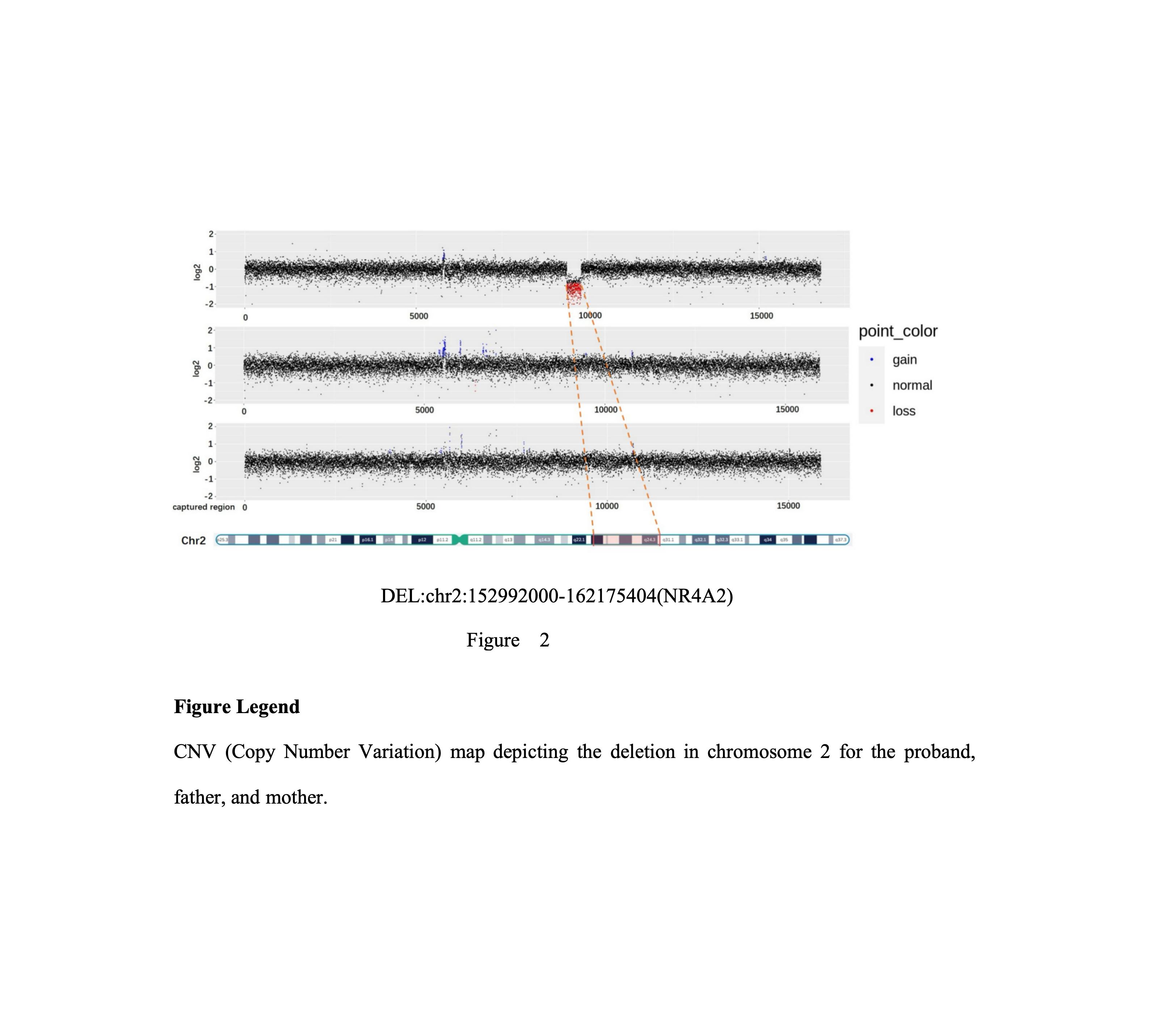

Supplement: Supplementary file 1 [file DataSheet1.zip › Supplementary files/Figure 2.jpg]

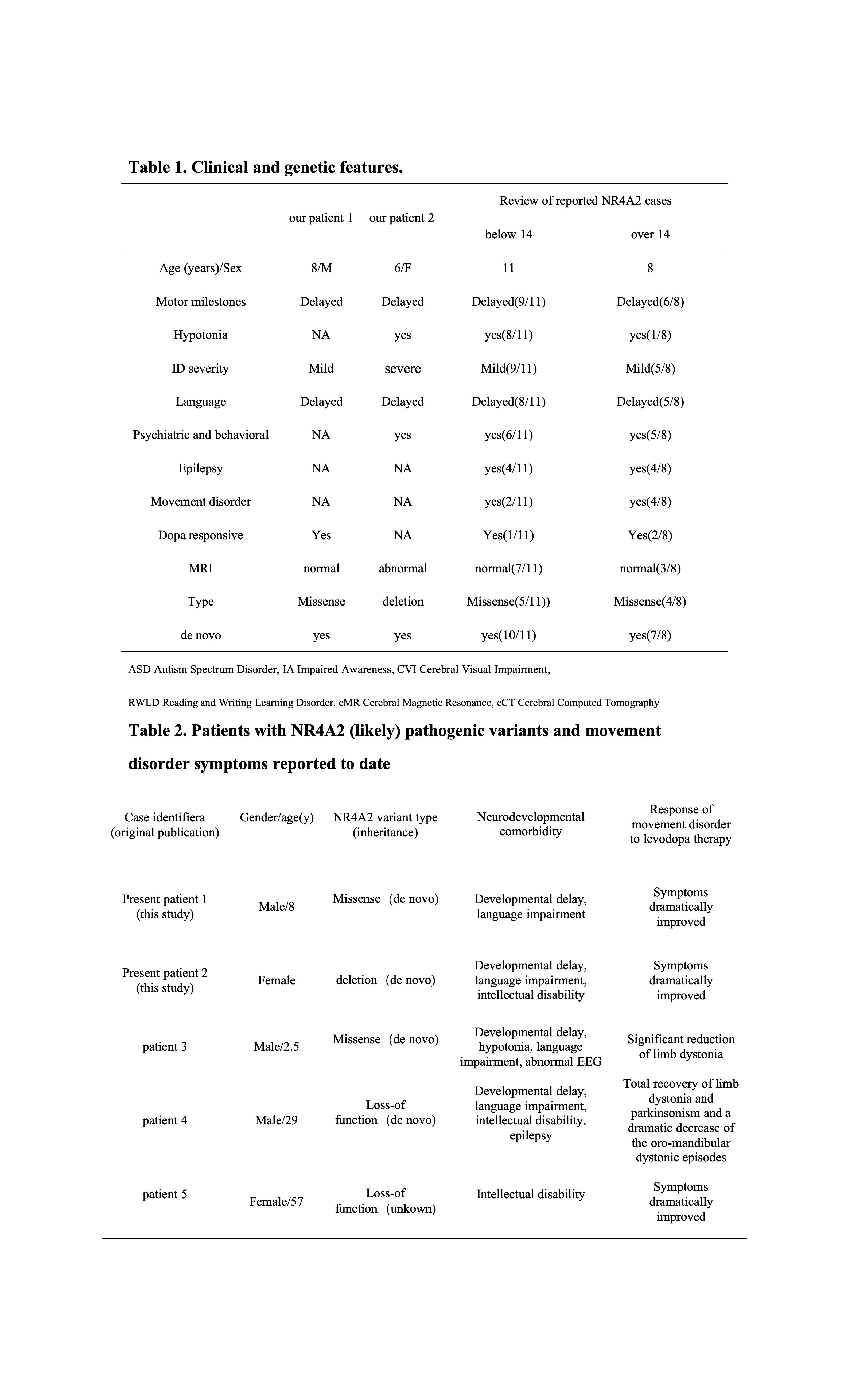

Supplement: Supplementary file 1 [file DataSheet1.zip › Supplementary files/table.jpg]
